# Management of alcohol withdrawal syndrome in patients with alcohol-associated liver disease

**DOI:** 10.1097/HC9.0000000000000372

**Published:** 2024-01-22

**Authors:** Jessica A. Ratner, Hanna Blaney, Darius A. Rastegar

**Affiliations:** 1 Division of Addiction Medicine, Johns Hopkins School of Medicine, Baltimore, Maryland, USA; 2 Division of Gastroenterology and Hepatology, University of Maryland School of Medicine, Baltimore, Maryland, USA

## Abstract

Alcohol-associated liver disease is a common and severe sequela of excessive alcohol use; effective treatment requires attention to both liver disease and underlying alcohol use disorder (AUD). Alcohol withdrawal syndrome (AWS) can be dangerous, is a common barrier to AUD recovery, and may complicate inpatient admissions for liver-related complications. Hepatologists can address these comorbid conditions by learning to accurately stage alcohol-associated liver disease, identify AUD using standardized screening tools (eg, Alcohol Use Disorder Identification Test), and assess risk for and symptoms of AWS. Depending on the severity, alcohol withdrawal often merits admission to a monitored setting, where symptom-triggered administration of benzodiazepines based on standardized scoring protocols is often the most effective approach to management. For patients with severe liver disease, selection of benzodiazepines with less dependence on hepatic metabolism (eg, lorazepam) is advisable. Severe alcohol withdrawal often requires a “front-loaded” approach with higher dosing, as well as intensive monitoring. Distinguishing between alcohol withdrawal delirium and HE is important, though it can be difficult, and can be guided by differentiating clinical characteristics, including time to onset and activity level. There is little data on the use of adjuvant medications, including anticonvulsants, dexmedetomidine, or propofol, in this patient population. Beyond the treatment of AWS, inpatient admission and outpatient hepatology visits offer opportunities to engage in planning for ongoing management of AUD, including initiation of medications for AUD and referral to additional recovery supports. Hepatologists trained to identify AUD, alcohol-associated liver disease, and risk for AWS can proactively address these issues, ensuring that patients’ AWS is managed safely and effectively and supporting planning for long-term recovery.

## INTRODUCTION

Alcohol-associated liver disease (ALD) is a common and severe complication of excessive alcohol use, with up to one-third of patients with alcohol use disorder (AUD) going on to develop various forms of ALD.^1,2^

Alcohol consumption is common in the United States, with 67% of adults reporting past month use. Alcohol use is more prevalent among men, with 69% of men reporting any alcohol use in the past year compared to 65% of women.^3^ Hazardous alcohol use and binge drinking are increasing in the United States, leading to increased morbidity and mortality.^4^ An analysis of death certificates from 2019 to 2020 demonstrated a 25.5% increase in deaths involving alcohol, with a 22.4% increase in yearly deaths from ALD from 24,106 to 29,504.^5^ Given the high prevalence of alcohol use in the United States, it is not surprising that 11.3% of people 18 years and older met diagnostic criteria for AUD in 2021, with men having a higher prevalence (13.2%) than women (9.5%).^3^

Alcohol is the leading cause of cirrhosis in many high-income countries, accounting for half of all cirrhosis-related deaths worldwide.^1^ Alcohol use has a dose-response effect on the risk of liver disease. In a systematic review, increasing alcohol consumption, measured in grams per day, was associated with an increased relative risk for cirrhosis.^6^ ALD remains the most common diagnosis for men referred for liver transplant and the second most common cause (after metabolic dysfunction–associated liver disease) for women in the US.^7^

Given that many patients with ALD also have AUD, the hepatologist must be comfortable and competent in managing both disorders. As abstinence from alcohol has been shown to improve morbidity and mortality at all stages of ALD, it is necessary to treat both the ALD and the underlying AUD.^8,9^ In fact, pharmacotherapy for AUD has been shown to reduce the incidence and progression of ALD and improve survival.^10,11^ Thus, treating only liver disease increases the risk of treatment failure, with transient improvements reversed if the patient returns to alcohol use.

Many patients with AUD have symptoms of withdrawal with cessation of alcohol intake—alcohol withdrawal syndrome (AWS) management is thus an important component of early management for those patients seeking AUD treatment. AWS can also develop when a patient’s alcohol consumption is abruptly decreased due to circumstances that limit access to alcohol. For example, many patients with ALD who are actively drinking require hospital admission for decompensation or alcohol-associated hepatitis (AH). As such, many of these patients will experience AWS while admitted. Thus, the hepatologist must be familiar with both the diagnosis and treatment of AWS in both inpatient and outpatient settings.

The estimated prevalence of AWS varies widely depending on the definition used and the population studied. In a national population-based survey conducted in 2012–2013, 14% of those with past-year unhealthy alcohol use (about 1/3 of respondents) reported having had alcohol withdrawal symptoms in the past year, comprising 5% of the total sample.^12^ Among hospitalized patients, estimates for the incidence of AWS range from 1% to 5%.^13–15^ While estimated rates of delirium tremens and alcohol withdrawal seizures are similarly variable, several studies suggest that 3%–11% of inpatients with AWS develop one of these complications.^16–18^

The rates of AWS are undoubtedly higher among inpatients treated by hepatologists–in one national sample of patients at Veterans Administration hospitals, 14% of patients admitted for liver injury developed AWS, and the presence of cirrhosis was associated with an increased probability of developing AWS during inpatient stays. Among hospitalized patients, more than one-fourth with prior-year AUD and more than two-thirds with prior-year AWS developed AWS during their hospital stay.^14^ In another study, 32% of patients admitted with AH developed AWS during their hospitalization.^19^

This article aims to support the hepatologist in the diagnosis of AUD and the identification and management of AWS among patients with ALD, as well as provide strategies for initiating ongoing AUD treatment. To conduct this review, we searched PubMed in July 2023 using terms including “alcohol withdrawal,” “liver disease,” “cirrhosis,” “alcohol use disorder,” and “treatment.” Results were reviewed for relevant citations, and additional references were identified through the review of these articles.

### Identifying alcohol use disorder

AUD is a chronic medical condition characterized by continued alcohol use despite adverse consequences. The 11 Diagnostic and Statistical Manual of Mental Disorders-5 criteria for diagnosis of AUD (which replaced Diagnostic and Statistical Manual of Mental Disorders-IV diagnoses “alcohol abuse,” and “alcohol dependence”) can be grouped into 4 categories: (1) impaired control, (2) social impairment, (3) risky use, and (4) pharmacologic dependence. The severity of AUD is determined by the number of criteria endorsed, with 2–3 symptoms defining mild AUD, 4–5 symptoms defining moderate AUD, and 6 or more symptoms defining severe AUD.^20^

Professional organizations, including the American Association for the Study of Liver Diseases, recommend validated screening tools to identify problematic alcohol use.^21^ Though no tools have been specifically validated for patients with ALD, the Alcohol Use Disorder Identification Test (AUDIT) is a 10-item tool with strong performance in the general population, assessing consumption, loss of control, and adverse consequences related to alcohol (see Table 1).^22^ The AUDIT-C, a 3-item version, is widely used for screening in outpatient and inpatient settings; scores of 4–5 or more may indicate hazardous alcohol consumption.^23–26^

**TABLE 1:** Alcohol Use Disorders Identification Test (AUDIT)

How often do you have a drink containing alcohol?	• Never • Monthly or less • 2–4 times a month • 2–3 times a week • 4 or more times a week
How many standard drinks containing alcohol do you have on a typical day when drinking?	• 1 or 2 • 3 or 4 • 5 or 6 • 7 to 9 • 10 or more
How often do you have 6 or more drinks on one occasion?	• Never • Less than monthly • Monthly • Weekly • Daily or almost daily
During the past year, how often have you found that you were not able to stop drinking once you had started?	• Never • Less than monthly • Monthly • Weekly • Daily or almost daily
During the past year, how often have you failed to do what was normally expected of you because of drinking?	∉ Never ∉ Less than monthly ∉ Monthly ∉ Weekly ∉ Daily or almost daily
During the past year, how often have you needed a drink in the morning to get yourself going after a heavy drinking session?	∉ Never ∉ Less than monthly ∉ Monthly ∉ Weekly ∉ Daily or almost daily
During the past year, how often have you had a feeling of guilt or remorse after drinking?	∉ Never ∉ Less than monthly ∉ Monthly ∉ Weekly ∉ Daily or almost daily
During the past year, have you been unable to remember what happened the night before because you had been drinking?	∉ Never ∉ Less than monthly ∉ Monthly ∉ Weekly ∉ Daily or almost daily
Have you or someone else been injured as a result of your drinking?	∉ No ∉ Yes, but not in the past year ∉ Yes, during the past year
Has a relative or friend, doctor or other health worker been concerned about your drinking or suggested you cut down?	∉ No ∉ Yes, but not in the past year ∉ Yes, during the past year

*Notes:* Scoring: Questions 1-8 are scored from 0 to 4 (with the first response option scoring 0, second scoring 1, etc). Questions 9 and 10 are scored 0, 2, or 4 (with the first response option scoring 0, second score 2, etc).

A score of 8 or more associated with harmful or hazardous drinking. Moderate to severe AUD is indicated by a score of 13 or more in women and 15 or more in men.

Subscales:

AUDIT-C includes questions 1–3.

AUDIT-PC includes questions 1, 2, 4, 5, and 10.

As a WHO-approved instrument, the AUDIT is in the public domain.

Abbreviations: AUD, alcohol use disorder; AUDIT, Alcohol Use Disorder Identification Test; WHO, World Health Organization.

Laboratory tests can be used as adjuncts in the identification of alcohol use but have limitations. Many lab abnormalities associated with heavy alcohol use, including elevation of aminotransferases, gamma-glutamyl transferase, and bilirubin, macrocytic anemia, and thrombocytopenia, are often found in patients with liver disease irrespective of etiology.^27^ Carbohydrate-deficient transferrin can reflect heavy drinking within the past 2–3 weeks, but lacks sensitivity and specificity in severe liver disease.^28,29^ Direct products of alcohol metabolism tend to be less affected by liver disease. Blood alcohol concentration reflects acute alcohol consumption and, thus, if used inpatient, is most useful if collected upon hospital presentation. Direct alcohol biomarkers urine ethyl glucuronide (3-day detection window), urine ethyl sulfate (3-day window), and blood phosphatidylethanol (2–3 wk window) have relatively high sensitivity and specificity for identifying alcohol use in liver disease. However, they exhibit significant interindividual variability, and urine ethyl glucuronide and ethyl sulfate can be affected by both renal dysfunction and diuretic use.^21,29,30^ Phosphatidylethanol levels correlate with the amount of alcohol ingested in the prior 2–4 weeks, but interindividual variability limits exact quantification. More research is needed, but studies to date suggest phosphatidylethanol values of < 20 ng/mL are consistent with minimal alcohol consumption and > 210 ng/mL with chronically high levels of use.^31^ Importantly, the utility of these 3 biomarkers in inpatient clinical care may be limited by institutional availability and/or prolonged processing time. Additionally, false negatives and, less commonly, false positives are possible with all alcohol biomarkers. Patients should be informed before testing, and results should be used as a prompt for further conversation with patients and to inform diagnosis rather than being considered diagnostic on their own.

Regardless of the tools used, clinicians should adopt a nonjudgmental, collaborative approach to help combat stigma and shame that often limit patient disclosure and help-seeking.^32^ Positive screening or laboratory testing should prompt further exploration of patterns and adverse consequences related to alcohol use to aid in the diagnosis of AUD and identify the need for AWS monitoring and longer-term AUD treatment.

### Stages of alcohol-associated liver disease

**FIGURE 1:**
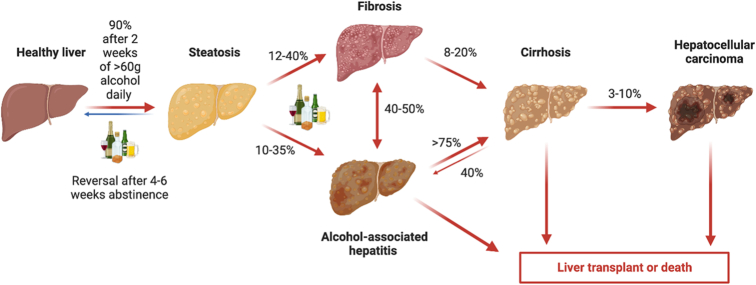
Alcohol-associated liver disease spectrum and natural history. With sustained heavy alcohol use, 90% of patients with normal livers will develop steatosis, which reverses after 4–6 weeks of abstinence. With continued alcohol use, patients can develop fibrosis or alcohol-associated steatosis, which may progress to cirrhosis. Patients with cirrhosis can develop HCC or end-stage liver disease necessitating liver transplantation.

ALD is a spectrum of disease, spanning from mild steatosis to decompensated cirrhosis and HCC (see Figure 1). Hepatic steatosis occurs in 90%–95% of patients with chronic, heavy alcohol use due to changes in the lipid synthetic pathway. A minority of individuals with heavy alcohol use will go on to experience alcohol-associated steatohepatitis. Persistent steatohepatitis will lead to the development of fibrosis in 20%–40% of patients, with a portion of these patients eventually developing cirrhosis, portal hypertension, decompensated liver disease, and HCC. Approximately 20%–30% of patients with ALD experience an episode of AH, an inflammatory process manifesting as rapid onset jaundice with features of liver failure. Severe AH carries a high mortality risk, with a 3-month mortality of 30%.^33,34^

### Identifying cirrhosis

While not all patients with ALD go on to develop cirrhosis, identifying cirrhosis is pertinent as these patients are at risk for clinically significant portal hypertension and decompensation, need screening for HCC, and should be considered for liver transplantation. Diagnosis of cirrhosis can be clinically challenging, with cirrhosis frequently diagnosed at the time of decompensation. For ALD, the odds of late diagnosis are 12 times higher than for viral hepatitis.^35^

History and physical examination can assist in the diagnosis of cirrhosis. Many patients experience muscle cramps, disordered sleep, and sexual dysfunction; however, these symptoms are neither sensitive nor specific. Exam findings such as palmar erythema, Terry nails, caput medusa, telangiectasia, gynecomastia, and ascites can also be suggestive of cirrhosis but have poor sensitivity.^36^

Laboratory values such as elevated transaminases and bilirubin, elevated INR, low albumin, and thrombocytopenia can assist in the diagnosis of cirrhosis. However, patients may have normal chemistry profiles. The platelet count is the earliest indicator of cirrhosis among the routine blood tests, with a platelet count threshold of less than 160 ×103 /µL having the highest diagnostic accuracy for cirrhosis.^36^ However, patients with AUD may have thrombocytopenia from direct toxicity from alcohol, including myelosuppression and accelerated platelet degradation.^37^ Routine abdominal imaging may suggest cirrhosis by revealing a nodular appearing liver or signs of portal hypertension, including ascites, splenomegaly, or the presence of collateral vessels, including evidence of varices. However, routine imaging has limited diagnostic accuracy and sensitivity for cirrhosis, with poor interrater reliability among radiologists.^38,39^

Liver biopsy was previously the gold standard for the diagnosis and assessment of steatosis and fibrosis; however, this modality is being replaced with noninvasive testing, including blood-based tests and elastography. In current practice, liver biopsy is reserved for situations where noninvasive testing is inconclusive or when the underlying etiology of liver disease is unclear.

Several laboratory-based scores have been developed to predict the presence of cirrhosis, though these methods cannot differentiate stages of fibrosis. Many of these scores use routinely available laboratory tests to risk stratify patients for having advanced fibrosis, such as the fibrosis-4 index (FIB-4, which uses age, alanine aminotransferase, aspartate aminotransferase, and platelet count). In the outpatient setting, following an elevated FIB-4 test, elastography can increase the probability of cirrhosis to 89% or greater.^40^ Using vibration-controlled transient elastography such as fibroscan, a liver stiffness measurement (LSM) of 15 kPa can identify cirrhosis with 95.5% specificity.^41^

However, both laboratory-based scores and elastography can be confounded in patients with heavy alcohol use. For example, all indices used to calculate the FIB-4 except age can be altered after acute alcohol use, with patients frequently having both elevated transaminases from alcohol use as well as thrombocytopenia. Likewise, alcohol induces hepatocyte swelling and inflammation, which can transiently increase LSM. It has been well documented that LSM significantly decreases after a period of abstinence. Thus, LSM is less useful in patients with recent alcohol use.^42^ Given that common noninvasive tests to identify fibrosis and cirrhosis are confounded by acute alcohol ingestion, accurately diagnosing cirrhosis in this population is challenging. While there is no official guidance on the diagnosis of chronic liver disease or cirrhosis in this unique population, greater reliance on imaging with attention towards the presence of collateral blood vessels or ascites may improve diagnostic accuracy. Additionally, despite the limitations in using FIB-4 or LSM to accurately diagnose chronic liver disease in patients who are actively drinking, if a patient does have the elevation of either test, there is some evidence to show that feedback on these results may be useful in educating patients on the deleterious effects of alcohol and may have a role in behavior change.^43^

### Alcohol withdrawal and severe liver disease

Alcohol withdrawal is a barrier to recovery from AUD and can be dangerous. Medically supervised withdrawal is a process to help individuals achieve abstinence and is sometimes a necessary first step in the process of recovery. The treatment of alcohol withdrawal is also an issue when individuals with AUD are hospitalized for other reasons, such as for decompensation of cirrhosis, and may not necessarily be seeking treatment for AUD. Epidemiologic data indicates that a minority of individuals with AUD seek treatment for this problem.^44^

### Alcohol withdrawal syndrome (AWS)

Alcohol exerts its effects on a wide variety of neurotransmitters. The main effects appear to be enhancement of the inhibitory neurotransmitter gamma-aminobutyric acid activity and inhibition of the excitatory neurotransmitter glutamate.^45^ When alcohol is abruptly withdrawn after regular use, gamma-aminobutyric acid activity is decreased, and glutamate is activated, leading to the clinical AWS.^46^

Alcohol withdrawal is generally divided into 3 clusters of symptoms^47^:

1. Autonomic hyperactivity—including tremulousness, sweating, tachycardia, nausea, vomiting, anxiety, and agitation; these symptoms typically appear within a few hours of the last drink and peak within 24–48 hours.
2. Neuronal excitation—including seizures; these typically appear within 12–48 hours of abstinence.
3. Alcohol withdrawal delirium (AWD; also referred to as “delirium tremens”)—including confusion, impaired consciousness, and hallucinations (visual, tactile, and occasionally auditory), along with severe autonomic hyperactivity; this typically occurs 48–72 hours after the last drink.

The Diagnostic and Statistical Manual of Mental Disorders-5 criteria for alcohol withdrawal requires the presence of at least 2 of the following 8 symptoms in the setting of cessation or reduction of alcohol consumption: (1) autonomic hyperactivity (sweating or tachycardia), (2) tremor, (3) insomnia, (4) nausea or vomiting, (5) transient visual, tactile, or auditory hallucinations or illusions, (6) psychomotor agitation, (7) anxiety, and (8) generalized tonic-clonic seizures.^20^

### Assessment of risk of withdrawal

Individuals with AUD may experience withdrawal symptoms that range from mild to life-threatening. Therefore, it is important to identify those at risk, particularly those at risk for severe withdrawal. One analysis of a national cohort of hospitalized veterans found that cirrhosis was a risk factor for AWS.^14^ A 2014 systematic review that included 15 studies found a number of factors that were associated with an increased risk of severe withdrawal, including prior delirium tremens or seizures, low potassium or platelet count, and elevated alanine transaminase or gamma-glutamyl transferase; however, a history of chronic liver disease was not a risk factor.^48^ While cirrhosis may not be a risk factor for severe withdrawal, it does appear to be a risk factor for mortality among those admitted for treatment of alcohol withdrawal.^49^

There are a number of tools that can be helpful for identifying individuals who are at risk for developing significant withdrawal. One of these is the AUDIT-PC (questions 1, 2, 4, 5, 10 of the AUDIT, see Table 1). In 1 study, a score of ≥ 4 on the AUDIT-PC had a sensitivity of 91% and specificity of 90% for identifying patients at risk for alcohol withdrawal.^50^ This study excluded patients who were admitted to an intensive care unit setting and did not report on medical comorbidities. Another tool is the 10-item Predictor of Alcohol Withdrawal Severity Score. In a study of 403 patients admitted to a general medical or surgical unit, a score of ≥ 4 on the Predictor of Alcohol Withdrawal Severity Score had a sensitivity of 93.1% and a specificity of 99.5% for identifying those at risk for moderate-severe withdrawal.^51^ In this study, 19% of the subjects had a primary diagnosis that was classified as “abdominal,” which included cirrhosis.

### Treatment setting

**FIGURE 2:**
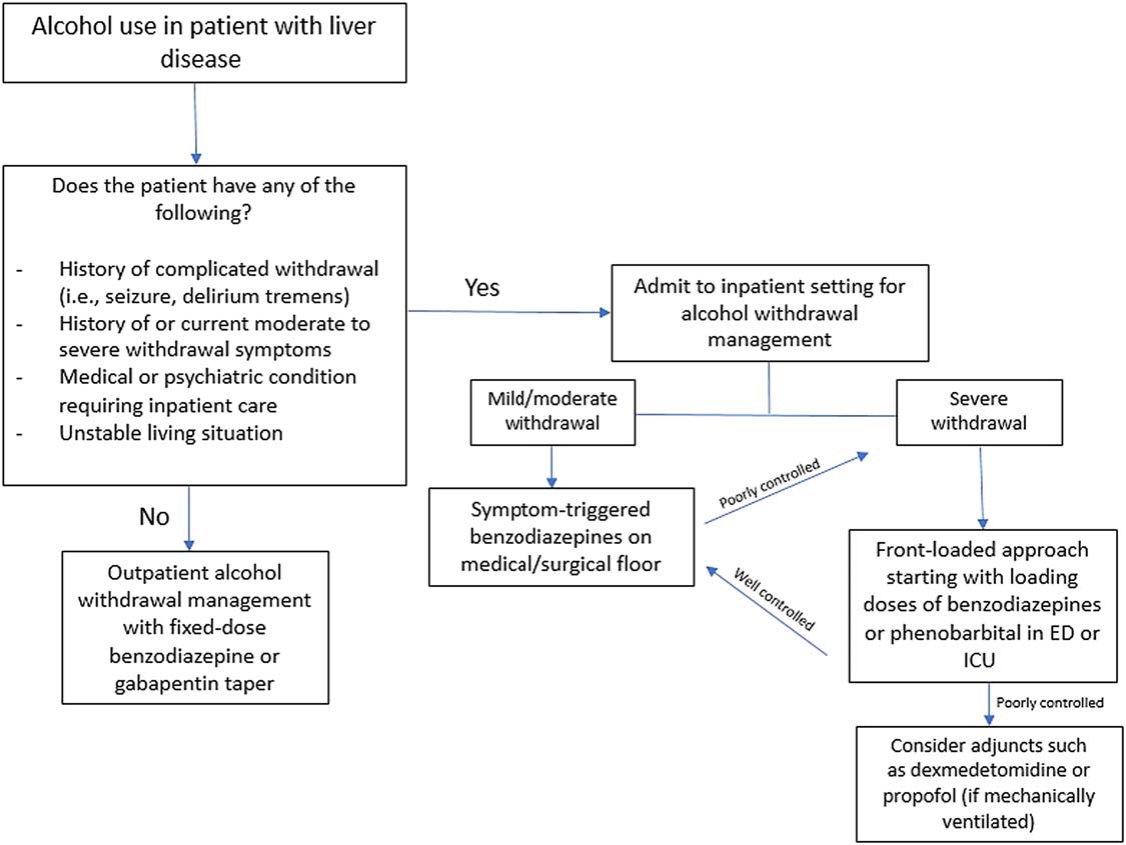
Selection of treatment setting and approach for alcohol withdrawal management. Abbreviations: ED, emergency department; ICU, intensive care unit.

For individuals who want to stop alcohol use and are not already hospitalized, a decision needs to be made regarding the treatment setting (see Figure 2). Many individuals with AUD can have their withdrawal managed as an outpatient.^52^ The choice between inpatient and outpatient treatment should be based on comorbid medical or psychiatric conditions and the severity of alcohol withdrawal, as well as the resources available.

For mildly symptomatic patients with a stable home environment and supportive family or friends, outpatient treatment with supervised daily visits to a treatment program or physician’s office is as effective as inpatient treatment.^53^ For moderately symptomatic patients, the choice of outpatient versus inpatient treatment should be based on what programs are available. Some outpatient programs are intensive, providing monitoring during the day while administering medication as needed and giving medication to take overnight when the patient returns home or to a shelter. Other outpatient programs may consist of brief daily visits with the assessment of withdrawal, administration of medication for withdrawal, and supplies of take-home medications for use later in the day and overnight.

Patients who are significantly symptomatic or who are at risk for severe withdrawal, including a prior history of withdrawal seizures or AWD, should, whenever possible, be admitted to a medically monitored inpatient unit for alcohol withdrawal management.

### Treatment of alcohol withdrawal

The standard (and best-studied) treatment for alcohol withdrawal is benzodiazepines, administered based on symptoms.^54^ For most individuals, 3–5 days of treatment is sufficient, but some may need longer, particularly those with severe withdrawal. Benzodiazepines can be given in a “fixed-dose” taper, “as-needed” depending on symptoms, or a combination of the 2. For outpatient treatment of a patient with a low risk for severe withdrawal, a fixed-dose taper with a long-acting agent like chlordiazepoxide is probably the best approach. For hospitalized patients at risk for severe withdrawal, providing a fixed-dose taper alone is insufficient and does not eliminate the need to monitor patients and provide as-needed dosing. Studies indicate that patients who are provided fixed-dose tapers in addition to as-needed medication receive more medication and have prolonged treatment courses without improvement in treatment outcomes.^55^ Thus, provided that adequate monitoring is possible (at least every 4 h for mild to moderate withdrawal, every 1–2 h for severe withdrawal), as-needed dosing based on symptoms is the best approach to inpatient treatment of AWS.

There is limited evidence that any particular benzodiazepine is most effective for the treatment of alcohol withdrawal.^56^ Likewise, there are no studies comparing the efficacy or safety of various agents among individuals with chronic liver disease. The selection of a particular agent depends on the pharmacokinetics (onset and duration of action), availability of oral versus parenteral formulations, and provider familiarity. In general, longer-acting agents like diazepam or chlordiazepoxide are preferred for the management of mild to moderate withdrawal because they require less frequent dosing. However, in patients with impaired liver function, there may be a risk of oversedation with some typically preferred agents. All benzodiazepines are metabolized in the liver. Chlordiazepoxide and diazepam are metabolized first through phase 1 metabolism with cytochrome P-450 oxidation and then through Phase 2 with gluconuridation. As such, active metabolites are produced, which can lead to a longer duration of action and increased risk of sedation. In contrast, lorazepam and oxazepam are primarily metabolized by Phase 2 metabolism with gluconuridation. In patients with liver disease, Phase 1 reactions involving cytochrome P-450 may be impaired, while glucuronidation remains unaffected. Thus, the metabolism of lorazepam and oxazepam is minimally affected by liver disease, and these agents may be preferred in this population.^57^ Accordingly, the American Society of Addiction Medicine (ASAM) guidelines recommend to “adjust medication dose or use medications with less dependence on hepatic metabolism.”^54^

For patients at risk for severe withdrawal or who are experiencing severe withdrawal (discussed in greater detail below), a “front-loaded” approach of boluses of medication works best.^58^ Such patients may initially require higher doses and frequency of dosing for treatment of their withdrawal (eg, 2–4 mg lorazepam, 10–20 mg of diazepam, or 50–100 of chlordiazepoxide every 1–2 h).

Withdrawal scales, most commonly the 10-item Clinical Institute Withdrawal Assessment-Alcohol revised scale,^59^ can be used to determine the need for doses; patients are generally dosed every 2–4 hours as long as their score is 8–10 or higher. However, the Clinical Institute Withdrawal Assessment-Alcohol revised relies on patient reports and subjective symptoms and is not useful for patients experiencing delirium. Shorter and more objective withdrawal scales have been developed that can be used as an alternative; one of these is the 5-item Brief Alcohol Withdrawal Scale (see Table 2).^60^ A sample symptom-triggered alcohol withdrawal protocol using benzodiazepines is provided in Table 3.

**TABLE 2:** Brief Alcohol Withdrawal Scale (BAWS)

	0 None	1 Mild	2 Moderate	3 Severe	Score
Tremor	No tremor	Not visible, but can be felt	Moderate, with arms extended	At rest, without arms extended	—
Diaphoresis/Sweats	No sweats	Mild, barely visible	Beads of sweat	Drenching sweats	—
Agitation	Alert and calm	Restless, anxious, apprehensive, movements not aggressive	Agitated, frequent nonpurposeful movement	Very agitated or combative, violent	—
Confusion/Orientation	Oriented to person, place, time	Disoriented to time (eg, by more than 2 d or wrong month or wrong year) or to place (eg, name of building, city, state), but not both	Disoriented to time and place	Disoriented to person	—
Hallucinations (visual, auditory, tactile)	None	Mild (vague report, reality testing intact)	Moderate (more defined hallucinations)	Severe (obviously responding to internal stimuli, poor reality testing)	—
TOTAL					—

Abbreviation: BAWS, Brief Alcohol Withdrawal Scale.

**TABLE 3:** Sample symptom-triggered alcohol withdrawal protocol

BAWS score	Treatment	Assessment interval
0–2	None	Reassess every 4–6 h
3–5	Lorazepam 2 mg orally every 4 h until BAWS < 3	Reassess every 4 h
6–8	Lorazepam 4 mg orally every 2 h until BAWS <6	Reassess every 2 h
8	Lorazepam 4 mg orally and notify the physician	Not applicable

Abbreviation: BAWS, Brief Alcohol Withdrawal Scale.

### Phenobarbital

Phenobarbital is a long-acting drug with effects on gamma-aminobutyric acid and glutamate. It can be used for the treatment of alcohol withdrawal, either alone or in combination with benzodiazepines. Some institutions use fixed-dose tapers of phenobarbital for alcohol withdrawal. Another strategy is a “front-loaded” approach providing a loading dose of phenobarbital and following that with as-needed benzodiazepines; this has been shown to lead to better outcomes than as-needed benzodiazepines alone.^61^ However, there is no evidence that phenobarbital is superior to providing comparable doses of benzodiazepines,^62^ and phenobarbital has a relatively narrow therapeutic window.^63^

### Adjunctive agents

A number of anticonvulsants have been studied for the treatment of alcohol withdrawal, including gabapentin, valproate, and carbamazepine. Gabapentin can be used to treat mild-moderate withdrawal in the outpatient setting.^64^ It is also sometimes used with as-needed benzodiazepines for more severe withdrawal in the inpatient setting, but this approach has not been shown to be superior to benzodiazepines alone.^65^ Gabapentin can also be continued after withdrawal treatment to prevent relapse but should be used with caution because of the sedating effect and the potential for misuse.^66,67^ The 2020 ASAM guidelines state that carbamazepine or gabapentin can be used for “patients experiencing mild withdrawal who are at minimal risk of developing severe or complicated withdrawal” and that “carbamazepine, gabapentin, or valproic acid (if no liver disease or childbearing potential) may be used as an adjunct to benzodiazepines.”^54^

Phenothiazines can be used alongside benzodiazepines for the treatment of delirium that is not responsive to benzodiazepines alone; however, they should not be used as monotherapy for alcohol withdrawal delirium.^54^

### Treatment of severe alcohol withdrawal

While a universally accepted definition is lacking, individuals who require admission to an intensive care unit for alcohol withdrawal or who experience seizures or delirium are considered to have “severe” or “complicated” alcohol withdrawal.^68^ Complications may be the result of withdrawal itself or medications given to treat withdrawal.

Treatment of severe alcohol withdrawal syndrome requires frequent assessment and high doses of sedatives—for this reason, it is best treated in an intensive care setting. As with less severe withdrawal, the treatment of choice is benzodiazepines, but these patients generally require higher doses that generally need to be administered intravenously at frequent intervals or by continuous infusion. Diazepam can be given in I.V. boluses, and lorazepam can also be given in I.V. boluses or as a continuous infusion. Phenobarbital can also be used as an adjunct for patients with difficult-to-control withdrawal symptoms. As noted earlier, the Clinical Institute Withdrawal Assessment-Alcohol revised is not useful for monitoring delirious patients, and a sedation-agitation scale (eg, Richmond Agitation Sedation Scale) can be used instead.

Anesthetic agents such as dexmedetomidine and propofol can help control the symptoms of alcohol withdrawal and decrease the need for benzodiazepines for patients in an intensive care unit setting. Dexmedetomidine, a selective alpha-2 adrenergic receptor agonist, can be used as an adjuvant to benzodiazepines for patients in an intensive care setting being treated for AWD, but there is no evidence that it is superior to adequately dosed benzodiazepines.^69^ It should not be used alone nor as initial therapy for alcohol withdrawal. Propofol is also used as an adjunct for the treatment of “resistant” severe alcohol withdrawal in patients treated in an intensive care unit, already requiring mechanical ventilation. However, there is insufficient evidence to support its efficacy as monotherapy or as an adjuvant compared to other treatments.^70^

A complicating factor is that for patients with chronic liver disease, it may be difficult to distinguish between AWD and HE. Although there is limited evidence to support this, there are a number of factors that may help distinguish between the two. One is the presence or absence of a history of recent alcohol use and the relationship of symptoms with the timing of the last drink, as withdrawal-related delirium often manifests 48–72 hours after the last alcohol intake (but may occur earlier); for this, it is often necessary to seek information from others. The delirium in HE is typically hypoactive, while patients with AWD typically are hyperactive and have dysautonomia with sweats.^71^ In addition, patients with alcohol withdrawal can have a rhythmic tremor in all extremities, while the tremor of HE is typically seen with outstretched arms and hands.

### Planning for ongoing AUD management

Medical complications of alcohol use can serve as a catalyst for change, and both outpatient hepatology visits and inpatient admission offer opportunities to engage in longer-term AUD treatment planning. Ongoing alcohol consumption, even in moderate amounts, has been linked to increased risk of liver disease decompensation and mortality.^72–75^ Thus, cessation of alcohol use is recommended for patients with liver disease—a goal that many patients with AUD find difficult to achieve without support.^21,73^

AUD treatment, which can include psychosocial interventions and medications, aims to help patients reduce/eliminate alcohol intake and repair aspects of their lives that have been harmed by alcohol use, such as relationships or employment. Psychosocial treatment options range from self-help/peer-led groups (for example, Alcoholics Anonymous) to outpatient individual or group counseling (eg, utilizing cognitive behavioral therapy or motivational enhancement therapy) to residential treatment.^76^ While there is limited data specifically focusing on populations with comorbid AUD and liver disease, data suggests greater effectiveness of integrated approaches that combine psychosocial interventions with medical care for this population.^77,78^ Selection of and referral to treatment should be guided by patient goals and preferences, as well as consideration of medical, mental health, and psychosocial circumstances that may affect the ability to engage in care.

Medications for AUD, often underutilized in patients with liver disease, can provide benefits and have been linked to reduced rates of hepatic decompensation and mortality among patients with cirrhosis.^10,11,79^ See Table 4 for details regarding several Food and Drug Administration-approved and off-label medications with evidence of benefit in the treatment of AUD. Given the lack of randomized controlled data in patients with known liver disease for most medications for AUD, the majority of the guidance in Table 4 is based on existing data in the general population, available observational data on liver disease, and expert opinion.

**TABLE 4:** Medications for alcohol use disorder

Medication	Considerations in patient selection	Starting dose	Titration recommendations	Metabolism	Common side effects
FDA-Approved for treatment of AUD					
Acamprosate	>No known safety concerns in liver disease >Avoid when CrCl ≤30 mL/min	CrCl > 50 mL/min: 666 mg PO TID CrCl 30–50 mL/min: 333 mg PO TID	No titration recommended	None	Diarrhea
Naltrexone	>Observational data suggestive of safety in compensated cirrhosis; limited data to guide use in decompensated cirrhosis or severe hepatitis >Avoid in patients receiving concurrent opioids (wait 7–10 d since last dose)	PO: 50 mg is the typical starting dose; consider 25 mg for patients previously receiving opioids or concerned about potential GI side effects IM: 380 mg monthly	If PO is started at 25 mg, the dosage can be increased to 50 mg after 1–3 d	Hepatic	Nausea, abdominal cramps/pain Headache Anxiety/nervousness Increased transaminases
Used off-label for treatment of AUD					
Baclofen	>Caution with HE due to risk of sedation >Caution with renal impairment	Variable starting doses across studies. 10 mg PO daily or 5 mg PO TID is reasonable. *Dose reduction is recommended with decreased CrCl.*	Variable titration approaches across studies. 10 mg PO TID is the minimum final dose in most studies. *Dose reduction is recommended with decreased CrCl.*	Minimal hepatic	Fatigue, dizziness, somnolence/sedation Dry mouth Paresthesia Muscle spasms/rigidity
Gabapentin	>Caution with HE due to risk of sedation >Caution with renal impairment >May have most benefit for patients who typically develop pronounced withdrawal symptoms	300 mg PO qHS *Dose reduction recommended with decreased CrCl.*	Day 2: 300 mg PO qAM + 300 mg qHS Day 3–4: 300 mg PO TID Day 5: 300 mg qAM and at noon+600 mg qHS *Dose reduction recommended with decreased CrCl.*	None	Dizziness, fatigue, somnolence Ataxia, nystagmus Peripheral edema Risk of non-medical use
Topiramate	>Avoid in patients at risk for HE given potential cognitive side effects >May have reduced clearance in liver disease	25 mg PO nightly *50% dose reduction for CrCl< 70 mL/min.*	Increase total daily dose by 25 mg per week to minimum effective dose (range 50–150 mg PO BID). *50% dose reduction for CrCl < 70 mL/min*	Minimal hepatic	Cognitive slowing/”brain fog” Dizziness, somnolence Paresthesias Loss of appetite

Note: Disulfiram is not included above, as it is not recommended for use in patients with liver disease due to reports of hepatoxicity.

Abbreviations: BID, twice daily; CrCl, creatinine clearance; IM, intramuscular; PO, by mouth; qAM, in the morning; qHS, at bedtime; TID, 3 times daily.

None of the Food and Drug Administration-approved medications have been formally studied in patients with liver disease. Among the Food and Drug Administration-approved options, acamprosate has the fewest safety concerns in liver disease; while data on effectiveness has been mixed,^80^ several systematic reviews have shown benefits in maintaining abstinence.^81–83^ Naltrexone has been found moderately effective in maintaining abstinence and reducing drinking^80–82^; however, early reports of hepatotoxicity have led to limited use in patients with liver disease. Drug levels may in fact be higher in patients with cirrhosis, particularly if decompensated^84^; however, research has not borne out concerns of hepatotoxicity,^85–88^ and a recent retrospective analysis among patients with liver disease suggests minimal hepatic risk.^89^ While further research is needed to guide the use of naltrexone in decompensated cirrhosis and severe hepatitis, a shared decision-making approach for those with less severe liver disease is appropriate.

Baclofen, used off-label for AUD, is the only medication with randomized trial data in patients with liver disease. While the research findings are somewhat mixed, they tend to support baclofen’s efficacy in maintaining abstinence, both within the general population and among patients with liver disease.^82,90–92^ Although minimal adverse effects have been observed across studies, it should be noted that studies generally exclude patients with HE, for whom potential sedating side effects could be more harmful.

Hepatologists should collaborate with addiction medicine providers, behavioral health clinicians, and social work to provide wrap-around support and improve the uptake of recovery services and medications for AUD.^93,94^ For patients admitted to the hospital, interdisciplinary teams can help identify and link patients with local resources to support AUD recovery after discharge. During prolonged hospitalizations, patients can also be encouraged to engage with recovery supports while still admitted, for example, through the use of tablets for participation in online mutual support groups (eg, Alcoholics Anonymous, Smart Recovery) or by working with peer recovery specialists, when available, who can provide one-on-one motivational support.^95^ When possible, medications for AUD should be initiated prior to discharge with the planned transition to a hepatology, primary care, or addiction medicine provider for ongoing management. Hepatology outpatient follow-up appointments offer an ongoing chance to check in with patients about recovery and provide continued nonjudgmental support—with particular attention to stability, AUD medication efficacy and side effects, and engagement with psychosocial treatments and additional recovery support at each outpatient visit.

## CONCLUSION

AUD and liver disease are comorbid conditions that require simultaneous management to effectively improve patient outcomes. AWS is a common barrier to AUD recovery and a frequent complication for patients hospitalized with liver-related decompensation. Hepatologists trained to identify AUD, ALD, and risk for AWS can proactively address these issues, ensuring that patients’ AWS is managed safely and effectively and offering patients the chance to begin treatment planning to support long-term AUD recovery.
